# Differential Use of Radiotherapy Fractionation Regimens in Prostate Cancer

**DOI:** 10.1001/jamanetworkopen.2023.37165

**Published:** 2023-10-10

**Authors:** Sarah A. Qureshy, Marshall A. Diven, Xiaoyue Ma, Ariel E. Marciscano, Jim C. Hu, Tim D. McClure, Christopher Barbieri, Himanshu Nagar

**Affiliations:** 1 currently a medical student at Weill Cornell Medicine, New York, New York; 2 New York Presbyterian-Brooklyn Methodist Hospital, Brooklyn, New York; 3 Department of Population Health Sciences, Division of Biostatistics, Weill Cornell Medicine, New York, New York; 4 Department of Radiation Oncology, Weill Cornell Medicine/NewYork-Presbyterian, New York, New York; 5 New York Presbyterian/Weill Cornell Medical Center, New York, New York; 6 Department of Urology, Weill Cornell Medicine, New York, New York

## Abstract

**Importance:**

Technical advances in treatment of prostate cancer and a better understanding of prostate cancer biology have allowed for hypofractionated treatment courses using a higher dose per fraction. Use of ultrahypofractionated stereotactic body radiotherapy (SBRT) has also been characterized.

**Objective:**

To characterize US national trends of different RT fractionation schemes across risk groups of prostate cancer.

**Design, Setting, and Participants:**

This retrospective cohort study used data collected by the National Cancer Database (NCDB) to characterize the fractionation regimens used for 302 035 patients diagnosed as having prostate cancer from January 1, 2004, to December 31, 2020, who underwent definitive RT. The analysis was performed between February 1 and April 30, 2023.

**Exposure:**

Stereotactic body RT or ultrahypofractionation, defined as 5 or fewer fractions of external beam RT (EBRT), moderate hypofractionation, defined as 20 to 28 fractions of EBRT, or conventional fractionation, defined as all remaining EBRT fractionation schemes.

**Main Outcomes and Measures:**

Temporal trends and clinical and sociodemographic factors associated with SBRT, moderate hypofractionation, and conventional fractionation use.

**Results:**

A total of 302 035 men receiving EBRT for localized prostate cancer between 2004 and 2020 were identified (40.1% aged 60-69 years). Black patients comprised 17.6% of this cohort; White patients, 77.9%; and other races and ethnicities, 4.5%. Patients with low-risk disease comprised 17.5% of the cohort; favorable intermediate-risk disease, 23.5%; unfavorable intermediate-risk disease, 23.9%; and high-risk disease, 35.1%. Treatment consisted of conventional fractionation for 81.2%, moderate hypofractionation for 12.9%, and SBRT for 6.0%. The rate of increase over time in patients receiving SBRT compared with conventional fractionation was higher (adjusted odds ratio [AOR] for 2005 vs 2004, 3.18 [95% CI, 2.04-4.94; *P* < .001]; AOR for 2020 vs 2004, 264.69 [95% CI, 179.33-390.68; *P* < .001]) than the rate of increase in patients receiving moderate hypofractionation compared with conventional fractionation (AOR for 2005 vs 2004, 1.05 [95% CI, 0.98-1.12; *P* = .19]; AOR for 2020 vs 2004, 4.41 [95% CI, 4.15-4.69; *P* < .001]). Compared with White patients, Black patients were less likely to receive SBRT compared with conventional fractionation or moderate hypofractionation (AOR for conventional fractionation, 0.84 [95% CI, 0.80-0.89; *P* < .001]; AOR for moderate hypofractionation, 0.77 [95% CI, 0.72-0.81; *P* < .001]). Compared with 2019, patients treated with all fractionation regimens declined in 2020 by 24.4%.

**Conclusions and Relevance:**

In this hospital-based cohort study of patients with prostate cancer treated with definitive EBRT, use of moderate hypofractionation and SBRT regimens for definitive prostate cancer treatment has increased from 2004 to 2020. Despite this increasing trend, findings suggest potential health care disparities for Black patients receiving EBRT for localized prostate cancer. The number of patients treated with EBRT in the year 2020 decreased, coinciding with official onset of the COVID-19 pandemic in March 2020.

## Key Points

**Question:**

Is there an association between clinical variables or sociodemographic patient characteristics and the radiotherapy regimen used for treatment of prostate cancer in the US?

**Findings:**

In a cohort study using data from 302 035 patients in the National Cancer Database, the use of stereotactic body radiotherapy (SBRT) for prostate cancer increased from 0.2% in 2004 to 12.4% in 2020. Older age, less severe disease, White race, living in an area with higher mean income, and living farther from the treatment center increased the chances of receiving SBRT.

**Meaning:**

These findings suggest that use of SBRT and moderately hypofractionated radiotherapy has increased over time but is also associated with several clinical and demographic patient factors.

## Teaser

This cohort study compares trends in the use of stereotactic body radiotherapy and moderate hypofractionation with conventional fractionation in the treatment of patients with localized prostate cancer.

## Introduction

Prostate cancer is the most diagnosed cancer and the second highest cause of cancer-related deaths among male patients in the US, with approximately 288 000 new cases and 34 700 deaths estimated in 2023.^1^ Initial management for newly diagnosed individuals with localized disease includes either active surveillance for patients on the lower end of the risk spectrum to definitive management with either prostatectomy or definitive radiotherapy (RT).^2^ For patients recommended to undergo definitive treatment, the preferred options include radical prostatectomy or RT, which may include external beam RT (EBRT) and/or brachytherapy. Within this study, 302 035 patients were identified using this inclusion and exclusion criteria shown in the included eFigure in Supplement 1.

Advances in RT technology and planning techniques such as intensity-modulated RT or volumetric arc therapy have led to more effective treatment plans that allow for adequate dose delivery while avoiding treatment-related toxic effects and off-target tissue effects.^3,4^ Along with technical advances, a greater understanding of prostate cancer biology has allowed for hypofractionated treatment courses using a higher dose per fraction. Studies^5,6,7,8,9^ have shown that moderately hypofractionated EBRT delivered in 2.5- to 3.0-Gy daily fractions over 4 to 6 weeks are characterized by toxicity profiles similar to those of conventional fractionation. As such, moderately hypofractionated RT is now the National Comprehensive Cancer Network (NCCN)–preferred regimen and is increasingly gaining traction in the US.

Another EBRT regimen for patients with localized prostate cancer is stereotactic body RT (SBRT), which is an ultrahypofractionated regimen delivering 6.0 to 9.5 Gy per fraction in 4 to 5 treatments. Recent publication of 2-year toxicity data from Prostate Advances in Comparative Evidence B, a phase 3 randomized clinical trial in patients with low- or intermediate-risk prostate cancer randomized to either conventional radiotherapy or SBRT,^10^ showed similar low rates of gastrointestinal tract and genitourinary Radiation Therapy Oncology Group toxic effects and no serious adverse events or treatment-related deaths. Indeed, trials comparing SBRT have shown this regimen to be a cost-effective alternative to conventionally fractionated EBRT or surgery and achieves comparable outcomes without added toxicity.^11,12,13,14,15^ As a result, the NCCN guidelines support SBRT use at facilities with appropriate infrastructure, as well as clinical and technical expertise.^2^ Mahase et al^16^ previously characterized US trends in the use of SBRT for prostate cancer. In this present study, we aim to compare trends in the use of SBRT and moderate hypofractionation with conventional fractionation in the treatment of localized prostate cancer.

## Methods

### Data Source

The National Cancer Database (NCDB) is now the largest clinical cancer registry in the world.^17^ The database captures approximately 70% of all patients newly diagnosed with cancer in the US. Because the patient data derived for this cohort study from the NCDB were deidentified, the need for institutional review and informed consent for patients was waived, consistent with the policies of Weill Cornell Medicine. This study followed the Strengthening the Reporting of Observational Studies in Epidemiology (STROBE) reporting guideline.

### Study Population

The NCDB was queried to identify patients diagnosed with localized prostate cancer from January 1, 2004, to December 31, 2020, who underwent definitive RT. A total of 2 012 367 patients were identified in this time frame. Stereotactic body RT (ultrahypofractionation) was defined as patients undergoing treatment with 4 or 5 fractions of EBRT. Moderate hypofractionation was defined as treatment with 20 to 28 fractions of EBRT. Conventional fractionation was defined as patients with the remaining fractionation schemes.

Prostate cancer diagnoses were made via biopsy of the primary site. The American Joint Committee on Cancer tumor category classification system was used to categorize tumor stage. The Gleason score was used to classify tumor grade (range: 6-10, with higher scores indicating higher grade of cancer). Patients with a single malignant primary tumor with invasive histologic findings were included in the analysis. Patients with missing demographic, clinical, and hospital information were excluded. Patients were excluded from analysis if they received alternative therapies, such as brachytherapy, or if they lacked sufficient data to classify their disease risk. Included patients received EBRT, including conformal or 3-dimensional conformal therapy, intensity-modulated RT, or stereotactic RT or radiosurgery (not otherwise specified, robotic, or gamma knife). Patients with tumor categories of cT4, lymph node involvement, or metastatic disease were excluded from analysis.

Patients with a tumor category of cT1a to cT2a, a Gleason score of 6, and a prostate-specific antigen (PSA) level less than 10 ng/mL (to convert to μg/L, multiply by 1.0) were defined as having low-risk disease. Patients with a tumor category of cT2b to cT2c, a Gleason score of 3 plus 4 or 4 plus 3, or a PSA level of 10 to 20 ng/mL were defined as having intermediate-risk disease. Patients with 1 intermediate-risk criterion were defined as having favorable intermediate-risk disease. Patients with a Gleason score of 4 plus 3 or with 2 or more intermediate-risk criteria were defined as having unfavorable intermediate-risk disease. Patients with tumor category cT3a to cT4, a Gleason score of 8 or higher, or a PSA level greater than 20 ng/mL were defined as having high-risk disease.^2^ The Charlson-Deyo Comorbidity Index was also included as a clinical variable. Several socioeconomic patient variables were also included for analysis, such as patient age, race and ethnicity, and insurance status and type. Race and ethnicity categories were used in multivariable analysis and were categorized according to NCDB codes, which included Black, White, and other race or ethnicity (which included Aleutian or Eskimo, American Indian, Asian Indian or Pakistani, Asian Indian, Chamorran, Chinese, Fiji Islander, Filipino, Guamanian, Hawaiian, Hmong, Japanese, Kampuchean, Korean, Laotian, Melanesian, Micronesian, New Guinean, Other Asian [including Asian and not otherwise specified], Pacific Islander, Pakistani, Polynesian, Samoan, Tahitian, Thai, Tongan, Vietnamese, not otherwise specified, other, and unknown). Treatment facility variables included US regional location and type of health care institution.

### Statistical Analysis

Data were analyzed from February 1 to April 30, 2023. Descriptive statistics were reported as frequency (percentage) for each categorical demographic, clinical, and hospital-level factor of interest. We performed a χ^2^ test to compare the univariate difference of factors between conventional fractionation, moderate hypofractionation, and ultrahypofractionation groups. Multivariate logistic regression was performed to examine the associations between the factors of interest and SBRT use, with adjusted odds ratios (AORs) reported. All tests were 2-sided and considered significant at a prespecified α level of .05. We used SAS, version 9.4 (SAS Institute Inc) for all analyses. GraphPad Prism, version 10.0.2 (GraphPad) was used to create the Figure plots.

## Results

Overall, 302 035 patients diagnosed with prostate cancer from 2004 to 2020 underwent definitive RT (14.8% aged <60 years, 40.1% aged 60-69 years, 39.0% aged 70-79 years, and 6.2% aged 80-89 years). Black patients comprised 17.6% of this cohort; White patients, 77.9%; and other race or ethnicity, 4.5% (including Aleutian or Eskimo, American Indian, Asian Indian or Pakistani, Asian Indian, Chamorran, Chinese, Fiji Islander, Filipino, Guamanian, Hawaiian, Hmong, Japanese, Kampuchean, Korean, Laotian, Melanesian, Micronesian, New Guinean, Other Asian [including Asian and not otherwise specified], Pacific Islander, Pakistani, Polynesian, Samoan, Tahitian, Thai, Tongan, Vietnamese, not otherwise specified, other, and unknown). The demographic and clinical characteristics of the patients are given in Table 1. A flow diagram outlining the selection of the patient cohort in the study is shown in the eFigure in Supplement 1. Of these patients, 17.5% were classified as having low-risk disease; 23.5%, favorable intermediate-risk disease; 23.9%, unfavorable intermediate-risk disease; and 35.1%, high-risk disease. Of all patients in the study, 81.2% were treated with conventional fractionation, 12.9% received moderate hypofractionation, and 6.0% received ultrahypofractionation (SBRT).

**Table 1.** Demographic and Clinical Characteristics of Patients Diagnosed With Prostate Cancer Between 2004 and 2020 Who Received Definitive RT^a^

Characteristic	Treatment group			
	All fractionation (n = 302 035)	SBRT (ultrahypofractionation) (n = 18 003)	Moderate hypofractionation (n = 38 932)	Conventional fractionation (n = 245 100)
Year of diagnosis				
2004	16 688 (5.5)	26 (0.1)	1730 (4.4)	14 932 (6.1)
2005	16 867 (5.6)	83 (0.5)	1822 (4.7)	14 962 (6.1)
2006	17 889 (5.9)	238 (1.3)	1767 (4.5)	15 884 (6.5)
2007	18 994 (6.3)	375 (2.1)	1914 (4.9)	16 705 (6.8)
2008	18 583 (6.2)	554 (3.2)	1737 (4.5)	16 292 (6.6)
2009	17 502 (5.8)	563 (3.1)	1520 (3.9)	15 419 (6.3)
2010	16 520 (5.5)	628 (3.6)	1406 (3.6)	14 486 (5.9)
2011	17 126 (5.7)	736 (4.1)	1404 (3.6)	14 986 (6.1)
2012	14 518 (4.8)	711 (3.9)	1197 (3.1)	12 610 (5.1)
2013	14 353 (4.8)	920 (5.1)	1159 (3.0)	12 274 (5.0)
2014	14 215 (4.7)	970 (5.4)	1187 (3.0)	12 058 (4.9)
2015	16 321 (5.4)	1109 (6.2)	1399 (3.6)	13 813 (5.6)
2016	18 573 (6.1)	1630 (9.1)	2082 (5.3)	14 861 (6.1)
2017	20 339 (6.7)	1974 (11.0)	3100 (8.0)	15 265 (6.2)
2018	22 059 (7.3)	2450 (13.6)	4207 (10.8)	15 402 (6.3)
2019	23 634 (7.8)	2831 (15.7)	5792 (14.9)	15 011 (6.1)
2020	17 854 (5.9)	2205 (12.2)	5509 (14.2)	10 140 (4.1)
Age, y				
<60	44 698 (14.8)	2759 (15.3)	6015 (15.5)	35 924 (14.7)
60-69	121 003 (40.1)	8192 (45.5)	16 495 (42.4)	96 316 (39.3)
70-79	117 707 (39.0)	6286 (34.9)	14 497 (37.2)	96 924 (39.5)
80-89	18 627 (6.2)	766 (4.3)	1925 (4.9)	15 936 (6.5)
Gleason score^b^				
<7	75 853 (25.1)	6214 (34.5)	6174 (15.9)	63 465 (25.9)
3 + 4 or 4 + 3	146 194 (48.4)	10 756 (59.7)	22 641 (58.2)	112 797 (46.0)
>7	79 988 (26.5)	1033 (5.7)	10 117 (26.0)	68 838 (28.1)
PSA level, ng/mL				
≤4	30 431 (10.1)	1899 (10.5)	3439 (8.8)	25 093 (10.2)
>4 to ≤10	171 549 (56.8)	12 573 (69.8)	22 713 (58.3)	136 263 (55.6)
>10 to ≤20	61 035 (20.2)	2708 (15.0)	8433 (21.7)	49 894 (20.4)
>20	39 020 (12.9)	823 (4.6)	4347 (11.2)	33 850 (13.8)
AJCC tumor stage				
T1a-T2a	240 067 (79.5)	16 620 (92.3)	30 681 (78.8)	192 766 (78.6)
T2b-T2c	48 803 (16.2)	1258 (7.0)	6542 (16.8)	41 003 (16.7)
T3a-T3b	13 165 (4.4)	125 (0.7)	1709 (4.4)	11 331 (4.6)
Charlson-Deyo Comorbidity Index score				
0	254 166 (84.2)	15 457 (85.9)	32 264 (82.9)	206 445 (84.2)
1	36 008 (11.9)	1986 (11.0)	4744 (12.2)	29 278 (11.9)
2	7805 (2.6)	356 (2.0)	1159 (3.0)	6290 (2.6)
≥3	4056 (1.3)	204 (1.1	765 (2.0)	3087 (1.3)
Race and ethnicity				
Black	53 275 (17.6)	2813 (15.6)	7602 (19.5)	42 860 (17.5)
White	235 201 (77.9)	14 331 (79.6)	29 441 (75.6)	191 429 (78.1)
Other^c^	13 559 (4.5)	859 (4.8)	1889 (4.9)	10 811 (4.4)
Primary payer				
Medicaid, Medicare, other government insurance	194 494 (64.4)	10 783 (59.9)	24 448 (62.8)	159 263 (65.0)
Private	98 668 (32.7)	6596 (36.6)	13 572 (34.9)	78 500 (32.0)
Uninsured	4118 (1.4)	150 (0.8)	435 (1.1)	3533 (1.4)
Unknown	4755 (1.6)	474 (2.6)	477 (1.2)	3804 (1.6)
Facility location				
New England	21 444 (7.1)	940 (5.2)	1939 (5.0)	18 565 (7.6)
Mid-Atlantic	56 518 (18.7)	6381 (35.4)	6668 (17.1)	43 469 (17.7)
South Atlantic	73 766 (24.2)	4035 (22.4)	10 380 (26.7)	59 351 (24.2)
Central				
Northeastern	52 001 (17.2)	2062 (11.5)	6847 (17.6)	43 092 (17.6)
Southeastern	17 237 (5.7)	971 (5.4)	2190 (5.6)	14 076 (5.7)
Northwestern	19 931 (6.6)	1047 (5.8)	2578 (6.6)	16 306 (6.7)
Southwestern	15 578 (5.2)	612 (3.4)	1648 (4.2)	13 318 (5.4)
Mountain	9665 (3.2)	677 (3.8)	1332 (3.4)	7656 (3.1)
Pacific Coast	35 895 (11.9)	1278 (7.1)	5350 (13.7)	29 267 (11.9)
Facility type				
Community cancer program	23 678 (7.8)	407 (2.3)	2832 (7.3)	20 439 (8.3)
Comprehensive community cancer program	126 601 (41.9)	5628 (31.3)	14 758 (37.9)	106 215 (43.3)
Academic or research program	96 977 (32.1)	8839 (49.1)	13 629 (35.0)	74 509 (30.4)
Integrated network cancer program	54 779 (18.1)	3129 (17.4)	7713 (19.8)	43 937 (17.9)
Median annual income, $				
<38 000	53 131 (17.6)	2153 (12.0)	6392 (16.4)	44 586 (18.2)
38 000-47 999	65 074 (21.5)	2587 (14.4)	7951 (20.4)	54 536 (22.3)
48 000-62 999	69 538 (23.0)	3509 (19.5)	9086 (23.3)	56 943 (23.2)
>62 999	114 292 (37.8)	9754 (54.2)	15 503 (39.8)	89 035 (36.3)
Educational level, % with no high school				
≥21	60 597 (20.1)	3078 (17.1)	7358 (18.9)	50 161 (20.5)
13.0-20.9	84 665 (28.0)	4614 (25.6)	10 598 (27.2)	69 453 (28.3)
7.0-12.9	88 307 (29.2)	5041 (28.0)	11 365 (29.2)	71 901 (29.3)
<7.0	68 466 (22.7)	5270 (29.3)	9611 (24.7)	53 585 (21.9)
Distance from facility, km				
<96	286 239 (94.8)	16 420 (91.2)	36 819 (94.6)	233 000 (95.1)
96-192	9118 (3.0)	1018 (5.7)	1419 (3.6)	6681 (2.7)
>192	6678 (2.2)	565 (3.1)	694 (1.8)	5419 (2.2)
Location				
Metropolitan	255 339 (84.5)	15 920 (88.4)	33 102 (85.0)	206 317 (84.2)
Urban	41 618 (13.8)	1875 (10.4)	5211 (13.4)	34 532 (14.1)
Rural	5078 (1.7)	208 (1.2)	619 (1.6)	4251 (1.7)

Abbreviations: AJCC, American Joint Committee on Cancer; RT, radiotherapy; PSA, prostate-specific antigen; SBRT, stereotactic body RT.

SI conversion factor: To convert PSA to μg/L, multiply by 1.0.

^a^ Data are presented as the No. (%) of patients. Percentages have been rounded and may not total 100.

^b^ Scores range from 6 to 10, with higher scores indicating higher grade of cancer.

^c^ Includes Aleutian or Eskimo, American Indian, Asian Indian or Pakistani, Asian Indian, Chamorran, Chinese, Fiji Islander, Filipino, Guamanian, Hawaiian, Hmong, Japanese, Kampuchean, Korean, Laotian, Melanesian, Micronesian, New Guinean, Other Asian (including Asian and not otherwise specified), Pacific Islander, Pakistani, Polynesian, Samoan, Tahitian, Thai, Tongan, Vietnamese, not otherwise specified, other, and unknown.

Use of SBRT increased from 0.2% in 2004 to 12.4% in 2020. Over time, the likelihood that patients received SBRT or moderate hypofractionation compared with conventional fractionation increased. The rate of increase over time in patients receiving SBRT compared with conventional fractionation was higher (AOR for 2005 vs 2004, 3.18 [95% CI, 2.04-4.94; *P* < .001]; AOR for 2020 vs 2004, 264.69 [95% CI, 179.33-390.68; *P* < .001]) than the rate of increase in patients receiving moderate hypofractionation compared with conventional fractionation (AOR for 2005 vs 2004, 1.05 [95% CI, 0.98-1.12; *P* = .19]; AOR for 2020 vs 2004, 4.41 [95% CI, 4.15-4.69]; *P* < .001). This is also shown in the Figure, as the use of moderate hypofractionation and ultrahypofractionation has increased over time. The likelihood of receiving SBRT compared with moderate hypofractionation also increased over time in the study (AOR for 2005 vs 2004, 3.17 [95% CI, 2.02-4.99; *P* < .001]; AOR for 2020 vs 2004, 40.81 [95% CI, 27.47-60.63; *P* < .001]). Data for multivariate logistic regression estimating SBRT vs moderate hypofractionation (Table 2), SBRT vs conventional fractionation (Table 3), and moderate hypofractionation vs conventional fractionation (Table 4) are presented herein.

**Figure.**
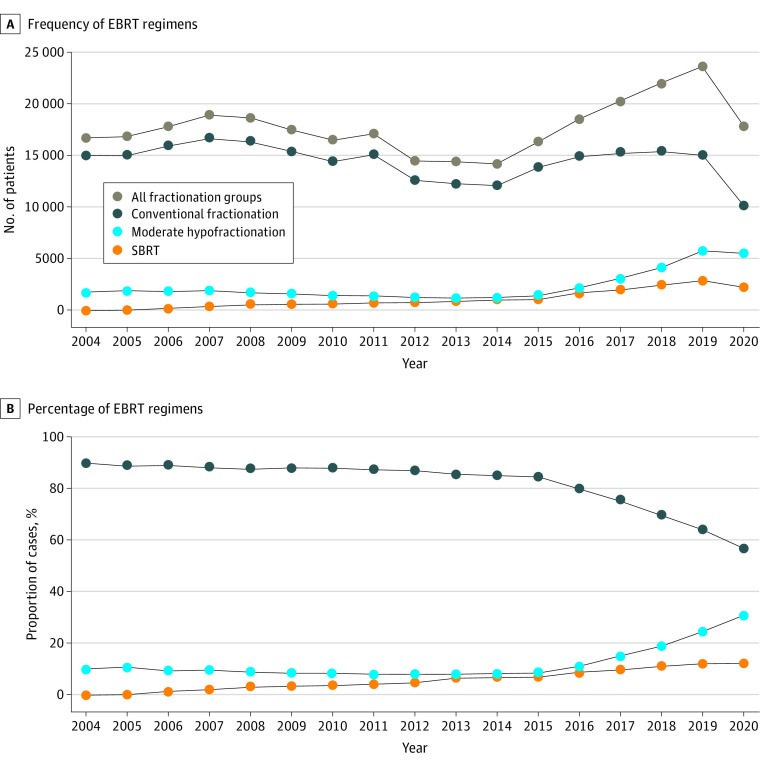
Frequency and Percentage of External Beam Radiotherapy (EBRT) Fractionation Regimens From 2004 to 2020 SBRT indicates stereotactic body radiotherapy.

**Table 2.** Multivariate Logistic Regression Model Estimating SBRT vs Moderate Hypofractionation Use Among 56 935 Patients^a^

Patient characteristic	AOR (95% CI)	*P* value
Year of diagnosis		
2004	1 [Reference]	NA
2005	3.17 (2.02-4.99)	<.001
2006	9.50 (6.26-14.43)	<.001
2007	12.62 (8.38-19.01)	<.001
2008	21.44 (14.29-32.16)	<.001
2009	26.53 (17.67-39.82)	<.001
2010	35.48 (23.65-53.23)	<.001
2011	39.01 (26.04-58.45)	<.001
2012	47.87 (31.91-71.81)	<.001
2013	65.30 (43.60-97.81)	<.001
2014	73.01 (48.76-109.30)	<.001
2015	71.94 (48.12-107.54)	<.001
2016	73.96 (49.63-110.22)	<.001
2017	59.49 (39.99-88.49)	<.001
2018	51.24 (34.49-76.13)	<.001
2019	43.99 (29.63-65.31)	<.001
2020	40.81 (27.47-60.63)	<.001
Age, y		
<60	1 [Reference]	NA
60-69	1.14 (1.07-1.22)	<.001
70-79	1.16 (1.08-1.25)	<.001
80-89	1.59 (1.42-1.79)	<.001
Gleason score^b^		
<7	1 [Reference]	NA
3 + 4 or 4 + 3	0.38 (0.36-0.40)	<.001
>7	0.09 (0.09-0.10)	<.001
PSA, ng/mL		
≤4	1 [Reference]	NA
>4 to ≤10	1.01 (0.94-1.08)	.81
>10 to ≤20	0.69 (0.64-0.75)	<.001
>20	0.55 (0.50-0.62)	<.001
AJCC tumor stage		
T1a-T2a	1 [Reference]	NA
T2b-T2c	0.53 (0.49-0.53)	<.001
T3a-T3b	0.21 (0.17-0.25)	<.001
Charlson-Deyo Comorbidity Index score		
0	1 [Reference]	NA
1	0.97 (0.91-1.04)	.37
2	0.77 (0.68-0.88)	<.001
≥3	0.62 (0.52-0.73)	<.001
Race and ethnicity		
White	1 [Reference]	NA
Black	0.77 (0.72-0.81)	<.001
Other^c^	0.92 (0.83-1.01)	.08
Primary payer		
Private	1 [Reference]	NA
Medicaid, Medicare, and other government insurance	1.05 (1.00-1.10)	.06
Uninsured	0.99 (0.80-1.22)	.91
Unknown	2.11 (1.81-2.46)	<.001
Facility location		
New England	1 [Reference]	NA
Mid-Atlantic	1.78 (1.62-1.96)	<.001
South Atlantic	0.89 (0.81-0.98)	.02
Central		
Northeastern	0.82 (0.74-0.91)	.001
Southeastern	1.18 (1.05-1.34)	.008
Northwestern	0.98 (0.87-1.10)	.68
Southwestern	1.09 (0.95-1.25)	.22
Mountain	1.29 (1.12-1.47)	<.001
Pacific Coast	0.51 (0.46-0.57)	<.001
Facility type		
Academic or research program	1 [Reference]	NA
Community cancer program	0.31 (0.27-0.35)	<.001
Comprehensive community cancer program	0.78 (0.74-0.82)	<.001
Integrated network cancer program	0.74 (0.70-0.79)	<.001
Median annual income, $		
>62 999	1 [Reference]	NA
48 000-62 999	0.60 (0.57-0.64)	<.001
38 000-47 999	0.48 (0.45-0.51)	<.001
<38 000	0.48 (0.44-0.52)	<.001
Educational level, % with no high school		
<7.0	1 [Reference]	NA
7.0-12.9	0.99 (0.93-1.05)	.69
13.0-20.9	1.23 (1.15-1.32)	<.001
≥21.0	1.43 (1.32-1.54)	<.001
Distance from facility, km		
<96	1 [Reference]	NA
96-192	1.93 (1.74-2.13)	<.001
>192	2.07 (1.82-2.36)	<.001
Location		
Metropolitan	1 [Reference]	NA
Urban	1.07 (1.00-1.15)	.06
Rural	0.94 (0.78-1.13)	.50

Abbreviations: AJCC, American Joint Committee on Cancer; AOR, adjusted odds ratio; NA, not applicable; PSA, prostate-specific antigen; SBRT, stereotactic body radiotherapy.

SI conversion factor: To convert PSA to μg/L, multiply by 1.0.

^a^ Includes 18 003 patients undergoing SBRT and 38 932 undergoing moderate fractionation.

^b^ Scores range from 6 to 10, with higher scores indicating higher grade of cancer.

^c^ Includes Aleutian or Eskimo, American Indian, Asian Indian or Pakistani, Asian Indian, Chamorran, Chinese, Fiji Islander, Filipino, Guamanian, Hawaiian, Hmong, Japanese, Kampuchean, Korean, Laotian, Melanesian, Micronesian, New Guinean, Other Asian (including Asian and not otherwise specified), Pacific Islander, Pakistani, Polynesian, Samoan, Tahitian, Thai, Tongan, Vietnamese, not otherwise specified, other, and unknown.

**Table 3.** Multivariate Logistic Regression Model Estimating SBRT vs Conventional Fractionation Use Among 263 103 Patients^a^

Patient characteristic	AOR (95% CI)	*P* value
Year of diagnosis		
2004	1 [Reference]	NA
2005	3.18 (2.04-4.94)	<.001
2006	8.58 (5.72-12.89)	<.001
2007	13.02 (8.73-19.40)	<.001
2008	20.40 (13.75-30.28)	<.001
2009	22.41 (15.10-33.25)	<.001
2010	28.93 (19.51-42.90)	<.001
2011	31.77 (21.45-47.07)	<.001
2012	39.88 (26.91-59.09)	<.001
2013	56.01 (37.85-82.88)	<.001
2014	67.45 (45.59-99.79)	<.001
2015	68.25 (46.17-100.90)	<.001
2016	97.45 (66.01-143.85)	<.001
2017	127.22 (86.22-187.72)	<.001
2018	159.04 (107.83-234.56)	<.001
2019	198.00 (134.27-291.98)	<.001
2020	264.69 (179.33-390.68)	<.001
Age, y		
<60	1 [Reference]	NA
60-69	1.10 (1.05-1.16)	<.001
70-79	1.02 (0.96-1.08)	.59
80-89	1.21 (1.10-1.33)	<.001
Gleason score^b^		
<7	1 [Reference]	NA
3 + 4 or 4 + 3	0.53 (0.51-0.56)	<.001
>7	0.09 (0.08-0.09)	<.001
PSA, ng/mL		
≤4	1 [Reference]	NA
>4 to ≤10	0.99 (0.94-1.05)	.83
>10 to ≤20	0.67 (0.63-0.72)	<.001
>20	0.41 (0.37-0.45)	<.001
AJCC tumor stage		
T1a-T2a	1 [Reference]	NA
T2b-T2c	0.61 (0.58-0.65)	<.001
T3a-T3b	0.21 (0.17-0.25)	<.001
Charlson-Deyo Comorbidity Index score		
0	1 [Reference]	NA
1	0.97 (0.92-1.03)	.33
2	0.74 (0.66-0.83)	<.001
≥3	0.68 (0.59-0.80)	<.001
Race and ethnicity		
White	1 [Reference]	NA
Black	0.84 (0.80-0.89)	<.001
Other^c^	0.89 (0.82-0.96)	.004
Primary payer		
Private	1 [Reference]	NA
Medicaid, Medicare, and other government insurance	0.94 (0.91-0.98)	.004
Uninsured	0.76 (0.63-0.90)	.002
Unknown	1.71 (1.53-1.91)	<.001
Facility location		
New England	1 [Reference]	NA
Mid-Atlantic	2.85 (2.64-3.07)	<.001
South Atlantic	1.59 (1.47-1.72)	<.001
Central		
Northeastern	1.20 (1.10-1.31)	<.001
Southeastern	1.99 (1.80-2.20)	<.001
Northwestern	1.70 (1.54-1.87)	<.001
Southwestern	0.85 (0.76-0.95)	.003
Mountain	2.04 (1.83-2.28)	<.001
Pacific Coast	0.89 (0.82-0.98)	.02
Facility type		
Academic or research program	1 [Reference]	NA
Community cancer program	0.21 (0.19-0.24)	<.001
Comprehensive community cancer program	0.52 (0.50-0.54)	<.001
Integrated network cancer program	0.75 (0.72-0.79)	<.001
Median annual income, $		
>62 999	1 [Reference]	NA
48 000-62 999	0.58 (0.56-0.61)	<.001
38 000-47 999	0.44 (0.41-0.46)	<.001
<38 000	0.40 (0.37-0.43)	<.001
Educational level, % with no high school		
<7.0	1 [Reference]	NA
7.0-12.9	0.91 (0.87-0.95)	<.001
13.0-20.9	1.13 (1.07-1.19)	<.001
≥21.0	1.28 (1.20-1.37)	<.001
Distance from facility, km		
<96	1 [Reference]	NA
96-190	2.40 (2.21-2.60)	<.001
>192	1.30 (1.18-1.44)	<.001
Location		
Metropolitan	1 [Reference]	NA
Urban	1.13 (1.07-1.20)	<.001
Rural	0.98 (0.84-1.15)	.81

Abbreviations: AJCC, American Joint Committee on Cancer; AOR, adjusted odds ratio; NA, not applicable; PSA, prostate-specific antigen; SBRT, stereotactic body radiotherapy.

SI conversion factor: To convert PSA to μg/L, multiply by 1.0.

^a^ Includes 18 003 patients undergoing SBRT and 245 100 undergoing conventional fractionation.

^b^ Scores range from 6 to 10, with higher scores indicating higher grade of cancer.

^c^ Includes Aleutian or Eskimo, American Indian, Asian Indian or Pakistani, Asian Indian, Chamorran, Chinese, Fiji Islander, Filipino, Guamanian, Hawaiian, Hmong, Japanese, Kampuchean, Korean, Laotian, Melanesian, Micronesian, New Guinean, Other Asian (including Asian and not otherwise specified), Pacific Islander, Pakistani, Polynesian, Samoan, Tahitian, Thai, Tongan, Vietnamese, not otherwise specified, other, and unknown.

**Table 4.** Multivariate Logistic Regression Model Estimating Moderate Hypofractionation vs Conventional Fractionation Use Among 284 032 Patients^a^

Patient characteristic	AOR (95% CI)	*P* value
Year of diagnosis		
2004	1 [Reference]	NA
2005	1.05 (0.98-1.12)	.19
2006	0.95 (0.88-1.02)	.13
2007	0.97 (0.90-1.03)	.31
2008	0.88 (0.82-0.95)	<.001
2009	0.80 (0.74-0.86)	<.001
2010	0.79 (0.73-0.85)	<.001
2011	0.75 (0.70-0.81)	<.001
2012	0.75 (0.69-0.81)	<.001
2013	0.74 (0.69-0.80)	<.001
2014	0.78 (0.72-0.85)	<.001
2015	0.80 (0.74-0.86)	<.001
2016	1.10 (1.03-1.18)	.007
2017	1.60 (1.50-1.70)	<.001
2018	2.17 (2.04-2.31)	<.001
2019	3.10 (2.92-3.30)	<.001
2020	4.41 (4.15-4.69)	<.001
Age, y		
<60	1 [Reference]	NA
60-69	0.99 (0.95-1.02)	.46
70-79	0.87 (0.83-0.90)	<.001
80-89	0.71 (0.66-0.75)	<.001
Gleason score^b^		
<7	1 [Reference]	NA
3 + 4 or 4 + 3	1.65 (1.60-1.70)	<.001
>7	1.20 (1.16-1.24)	<.001
PSA, ng/mL		
≤4	1 [Reference]	NA
>4 to ≤10	1.01 (0.97-1.05)	.69
>10 to ≤20	0.96 (0.92-1.01)	.09
>20	0.75 (0.72-0.79)	<.001
AJCC tumor stage		
T1a-T2a	1 [Reference]	NA
T2b-T2c	1.11 (1.08-1.12)	<.001
T3a-T3b	0.91 (0.86-0.96)	<.001
Charlson-Deyo Comorbidity Index score		
0	1 [Reference]	NA
1	1.01 (0.97-1.04)	.76
2	1.00 (0.94-1.07)	.97
≥3	1.06 (0.97-1.15)	.18
Race and ethnicity		
White	1 [Reference]	NA
Black	1.14 (1.11-1.18)	<.001
Other^c^	1.05 (1.00-1.11)	.08
Primary payer		
Private	1 [Reference]	NA
Medicaid, Medicare, or other government insurance	0.88 (0.86-0.91)	<.001
Uninsured	0.77 (0.69-0.85)	<.001
Unknown	0.80 (0.73-0.89)	<.001
Facility location		
New England	1 [Reference]	NA
Mid-Atlantic	1.60 (1.52-1.69)	<.001
South Atlantic	1.89 (1.79-1.99)	<.001
Central		
Northeastern	1.68 (1.59-1.77)	<.001
Southeastern	1.78 (1.66-1.90)	<.001
Northwestern	1.65 (1.54-1.76)	<.001
Southwestern	1.19 (1.11-1.28)	<.001
Mountain	1.65 (1.52-1.78)	<.001
Pacific Coast	1.92 (1.82-2.04)	<.001
Facility type		
Academic or research program	1 [Reference]	NA
Community cancer program	0.74 (0.71-0.78)	<.001
Comprehensive community cancer program	0.76 (0.74-0.78)	<.001
Integrated network cancer program	0.96 (0.93-0.99)	.01
Median annual income, $		
>62 999	1 [Reference]	NA
48 000-62 999	0.94 (0.91-0.97)	<.001
38 000-47 999	0.86 (0.83-0.89)	<.001
<38 000	0.84 (0.80-0.87)	<.001
Educational level, % with no high school		
<7.0	1 [Reference]	NA
7.0-12.9	0.91 (0.88-0.94)	<.001
13.0-20.9	0.91 (0.87-0.94)	<.001
≥21.0	0.92 (0.88-0.96)	<.001
Distance from facility, kms		
<96	1 [Reference]	NA
96-192	1.28 (1.20-1.36)	<.001
>192	0.73 (0.68-0.80)	<.001
Location		
Metropolitan	1 [Reference]	NA
Urban	1.07 (1.03-1.11)	<.001
Rural	1.03 (0.94-1.12)	.57

Abbreviations: AJCC, American Joint Committee on Cancer; AOR, adjusted odds ratio; NA, not applicable; PSA, prostate-specific antigen.

SI conversion factor: To convert PSA to μg/L, multiply by 1.0.

^a^ Includes 38 932 patients undergoing moderate fractionation and 245 100 undergoing conventional fractionation.

^b^ Scores range from 6 to 10, with higher scores indicating higher grade of cancer.

^c^ Includes Aleutian or Eskimo, American Indian, Asian Indian or Pakistani, Asian Indian, Chamorran, Chinese, Fiji Islander, Filipino, Guamanian, Hawaiian, Hmong, Japanese, Kampuchean, Korean, Laotian, Melanesian, Micronesian, New Guinean, Other Asian (including Asian and not otherwise specified), Pacific Islander, Pakistani, Polynesian, Samoan, Tahitian, Thai, Tongan, Vietnamese, not otherwise specified, other, and unknown.

Older patients were more likely to receive SBRT compared with conventional fractionation (AOR for 80-89 vs <60 years of age, 1.21 [95% CI, 1.10-1.33]; *P* < .001) or moderate hypofractionation (AOR for 80-89 vs <60 years of age, 1.59 [95% CI, 1.42-1.79; *P* < .001]) and less likely to receive moderate hypofractionation compared with conventional fractionation (AOR for 80-89 vs <60 years of age, 0.71 [95% CI, 0.66-0.75; *P* < .001]). The use of SBRT was less likely than conventional fractionation in patients with a higher Gleason score (AOR for >7 vs <7, 0.09 [95% CI, 0.08-0.09]; *P* < .001), higher PSA level (AOR for >20 vs ≤4 ng/mL, 0.41 [95% CI, 0.37-0.45]; *P* < .001), and higher tumor grade (AOR for T3a-T3b vs T1a-T2a, 0.21 [95% CI, 0.17-0.25]; *P* < .001). A higher Charlson-Deyo Comorbidity Index score also decreased the likelihood of receiving SBRT over conventional fractionation (AOR for ≥3 vs 0, 0.68 [95% CI, 0.59-0.80]; *P* < .001). Similarly, the use of SBRT was also less likely than moderate hypofractionation in patients with a higher Gleason score (AOR for >7 vs <7, 0.09 [95% CI, 0.09-0.10]; *P* < .001), higher PSA level (AOR for >20 vs ≤4 ng/mL, 0.55 [95% CI, 0.50-0.62]; *P* < .001), higher tumor grade (AOR for T3a-T3b vs T1a-T2a, 0.21 [95% CI, 0.17-0.25]; *P* < .001), and higher Charlson-Deyo Comorbidity Index score (AOR for ≥3 vs 0, 0.62 [95% CI, 0.52-0.73]; *P* < .001).

Compared with White patients, Black patients and patients identifying as any other race or ethnicity were less likely to receive SBRT compared with conventional fractionation (AOR for Black vs White race, 0.84 [95% CI, 0.80-0.89; *P* < .001]; AOR for Other vs White race, 0.89 [95% CI, 0.82-0.96; *P* = .004]). Black patients were also less likely than White patients to receive SBRT compared with moderate hypofractionation (AOR for Black vs White race, 0.77 [95% CI, 0.72- 0.81]; *P* < .001). However, Black patients were more likely to receive moderate hypofractionation compared with conventional fractionation (AOR for Black vs White race, 1.14 [95% CI, 1.11-1.18]; *P* < .001), while patients of other racial backgrounds were as likely to receive moderate hypofractionation as conventional fractionation (AOR for other vs White race, 1.05 [95% CI, 1.00-1.11]; *P* = .08).

Patients who had Medicare, Medicaid, or other government insurance were less likely to receive SBRT instead of conventional fractionation compared with those with private insurance (AOR, 0.94 [95% CI, 0.91-0.98]; *P* = .004), and uninsured patients had even lower likelihood of receiving SBRT compared with conventional fractionation when compared with patients with private insurance (AOR, 0.76 [95% CI, 0.63-0.90]; *P* = .002). There was no difference in likelihood of receiving SBRT compared with moderate hypofractionation in uninsured patients (AOR, 0.99 [95% CI, 0.80-1.22]; *P* = .91) or those with government insurance (AOR, 1.05 [95% CI, 1.00-1.10]; *P* = .06) when compared with privately insured patients. Patients were also less likely to receive moderate hypofractionation instead of conventional fractionation if they had government insurance (AOR, 0.88 [95% CI, 0.86-0.91]; *P* < .001) and even lower likelihood if they were uninsured (AOR, 0.77 [95% CI, 0.69-0.85]; *P* < .001) compared with patients with private insurance. Higher mean household income and lower mean educational level of the patient’s residential area increased the likelihood of receiving SBRT over either moderate hypofractionation (AOR for <$38 000 vs >$62 999, 0.48 [95% CI, 0.44- 0.52; *P* < .001]; AOR for ≥21.0 vs <7.0 years of education, 1.43 [95% CI, 1.32-1.54; *P* < .001]) or conventional fractionation (AOR for <$38 000 vs >$62 999, 0.40 [95% CI, 0.37-0.43; *P* < .001]; AOR for ≥21.0 vs <7.0 years of education, 1.28 [95% CI, 1.20-1.37; *P* < .001]).

Regarding location, patients receiving treatment at facilities located in the Mid-Atlantic (AOR, 2.85 [95% CI, 2.64-3.07]; *P* < .001), Mountain (AOR, 2.04 [95% CI, 1.83-2.28]; *P* < .001), southeastern Central (AOR, 1.99 [95% CI, 1.80-2.20]; *P* < .001), and northwestern Central (AOR, 1.70 [95% CI, 1.54-1.87]; *P* < .001) regions had a higher ratio of SBRT to conventional fractionation compared with New England. Conversely, patients treated in the southwestern Central and Pacific Coast regions saw a lower ratio of SBRT to conventional fractionation (AOR for southwestern Central, 0.85 [95% CI, 0.76-0.95; *P* = .003]; AOR for Pacific Coast, 0.89 [95% CI, 0.82-0.98; *P* = .02]) compared with those treated in New England. A higher ratio of SBRT to moderate hypofractionation was found in the Mid-Atlantic and Mountain regions compared with New England (AOR for Mid-Atlantic, 1.78 [95% CI, 1.62-1.96; *P* < .001]; AOR for Mountain, 1.29 [95% CI, 1.12-1.47; *P* < .001]); conversely, there was a lower frequency of SBRT compared with moderate hypofractionation in the Pacific Coast (AOR, 0.51 [95% CI, 0.46-0.57]; *P* < .001), northeastern Central (AOR, 0.82 [95% CI, 0.74-0.91]; *P* < .001), and South Atlantic (AOR, 0.89 [95% CI, 0.81-0.98]; *P* = .02) regions compared with New England.

The likelihood of receiving SBRT compared with both moderate hypofractionation (AOR, 0.31 [95% CI, 0.27-0.35]; *P* < .001) or conventional fractionation (AOR, 0.21 [95% CI, 0.19-0.24]; *P* < .001) was highest at academic or research program centers compared with community cancer centers. Patients were also less likely to be treated with SBRT compared with moderate hypofractionation at integrated network cancer network programs (AOR, 0.74 [95% CI, 0.70-0.79; *P* < .001) and comprehensive community cancer programs (AOR, 0.78 [95% CI, 0.74-0.82]; *P* < .001]) or to be treated with conventional fractionation at integrated network cancer programs (AOR, 0.75 [95% CI, 0.72-0.79; *P* < .001) or comprehensive community cancer programs (AOR, 0.52 [95% CI, 0.50-0.54; *P* < .001]) compared with academic or research institutions.

Increased distance between the treatment facility and a patient’s place of residence led to a higher likelihood of being treated with SBRT compared with either moderate hypofractionation (AOR, 2.07 [95% CI, 1.82-2.36]; *P* < .001) or conventional fractionation (AOR, 1.30 [95% CI, 1.18-1.44]; *P* < .001). When compared with metropolitan areas, patients in urban locations were as likely to receive SBRT as moderate hypofractionation (AOR, 1.07 [95% CI, 1.00-1.15]; *P* = .06) but more likely to receive SBRT than conventional fractionation (AOR, 1.13 [95% CI, 1.07-1.20]; *P* < .001). Patients in rural areas were as likely to receive SBRT as moderate hypofractionation (AOR, 0.94 [95% CI, 0.78-1.13]; *P* = .50) or conventional fractionation (AOR, 0.98 [95% CI, 0.84-1.15]; *P* = .81) when compared with patients in metropolitan areas. The likelihood of receiving moderate hypofractionation over conventional fractionation was higher in patients in urban areas (AOR, 1.07 [95% CI, 1.03-1.11]; *P* < .001) and similar in patients in rural areas (AOR, 1.03 [95% CI, 0.94-1.12]; *P* = .57) when compared with patients in metropolitan areas.

Due to the inclusion of 2020 data in the NCDB, we analyzed trends in the clinical, socioeconomic, and demographic data obtained during the SARS-CoV-2 pandemic. From 2019 to 2020, there was a 24.4% reduction in the number of patients treated with all fractionation regimens. Conventional fractionation saw a steeper decline (32.4%) than SBRT (22.1%) and moderate hypofractionation (4.8%) with year over year decline. Additionally, there was a trend showing an increase in representation of patients with higher Gleason scores as well as those with unfavorable intermediate-risk disease treated with RT. Interpretation of the included data from 2020 should be taken with caution due to potential external compounding factors related to unique challenges brought on by the COVID-19 pandemic.

## Discussion

Using a hospital-based cohort of patients with diagnoses of prostate cancer, we provide a descriptive analysis exploring trends in the use of SBRT and moderately hypofractionated RT compared with conventional RT. Our analyses included 302 035 patients receiving EBRT, with 81.2% treated using conventional fractionation, 12.9% with moderate hypofractionation, and 6.0% with SBRT. The proportion of patients receiving SBRT or moderate hypofractionation has increased from 2004 to 2020 while those receiving conventional fractionation decreased.

Stereotactic body RT was less likely to be used compared with either moderate hypofractionation and conventional fractionation in the treatment of patients with higher Gleason score, higher PSA level, higher tumor grade, and a higher Charlson-Deyo Comorbidity Index score. This suggests that patients with worse prognosis for primary prostate cancer were more likely to receive conventional fractionation, supporting our findings regarding the positive trend of SBRT use in the US from 2010 to 2015.^16^

Socioeconomic and demographic factors of patients were also associated with the fractionation schemes used over time. Older patients were more likely to receive SBRT compared with either moderate hypofractionation or conventional fractionation but were less likely to receive moderate hypofractionation compared with conventional fractionation. Black patients and patients of other races or ethnicities were less likely to receive SBRT compared with conventional fractionation, and Black patients were also less likely to receive SBRT compared with moderate hypofractionation. Black patients were more likely to receive moderate hypofractionation compared with conventional fractionation, and those identifying as other race or ethnicity were as likely to receive moderate hypofractionation as conventional fractionation. Living in an area with a higher mean income or lower mean level of education also increased the odds of being treated with SBRT compared with both moderate hypofractionation and conventional fractionation. Being uninsured or having public forms of insurance, as opposed to having private insurance, decreased the likelihood of being treated with both SBRT and moderate hypofractionation compared with conventional fractionation. Taken together, these findings suggest potential health disparities in certain patient populations, warranting further investigation.

The current lack of widespread access to SBRT is also illustrated through differences in the trend of ratios of RT regimens seen across US regions. Compared with all other areas of the US, patients treated in the Mid-Atlantic and Mountain regions had an increased likelihood of receiving SBRT compared with both moderate hypofractionation or conventional fractionation regimens. The ratio of SBRT to other regimens was highest at academic or research facilities and was lowest at community cancer centers. Stereotactic body RT is a newer modality of treatment requiring specialized equipment, training, and additional preparation, which could possibly explain the higher utilization rates by academic and research institutions. Living farther from the treatment facility increased patients’ likelihood of receiving SBRT. This is supportive of the potential advantage and added convenience of treatment with fewer visits with SBRT compared with moderate hypofractionation or conventional fractionation. Despite the increased likelihood associated with receiving SBRT treatment, conventional and moderately hypofractionated regimens are more common at all institutions and regions in the US.

In 2020, fewer patients were treated with RT across all treatment groups, likely reflecting a year of delayed diagnosis and definitive treatment in patients brought on by the COVID-19 pandemic. The increase in use of RT to treat higher-severity disease during the pandemic as well the greater percentage decrease in patients treated with conventional fractionation was likely due to compounding factors. Our findings from 2020 should be interpreted with caution and within the context of a period when treatment of patients with prostate cancer in the US was disrupted by external policies and measures aimed at limiting the spread of a deadly infectious disease. It will be interesting to investigate whether the pandemic accelerated the decline of conventional fractionation for patients with localized prostate cancer or if we may see resurgence in conventional fractionation following the pandemic. Given NCCN recommendations supporting moderate hypofractionation as the preferred regimen since 2018, conventional fractionation may well continue to decline in the future in lieu of shorter treatment courses.

### Limitations

This study has some limitations. First, it is subject to inherent biases based on its retrospective design. Second, the data are from the NCDB, which include an estimated 70% of all new cancer diagnoses^18,19^ and thereby limit the ability of the study to capture close to the entire population of patients with prostate cancer. Third, the NCDB does not include more specific details regarding RT. Further studies are needed to extrapolate the effects of different factors on RT fractionation schemes, such as image guidance, use of a rectal balloon, spacers or fiducials, treatment platforms, and whether spacing treatment fractions.

## Conclusions

The use of SBRT for prostate cancer treatment in the US increased between 2004 and 2020, although it was still used less frequently than moderate hypofractionation or conventional fractionation. Our cohort study illustrates the increased use of both SBRT and moderate hypofractionation regimens over time. Stereotactic body RT has been adopted for the treatment of prostate cancer predominantly in cases of less severe disease and in older patients. Patients treated with SBRT are more likely to live in areas with higher mean income, have private insurance, and be treated at academic centers, demonstrating the need for greater access to the treatment modality. During the pandemic, fewer patients with prostate cancer in the US underwent RT across all fractionation regimens. Patient access to SBRT would be increased with the adoption of the regimen by different types of health care institutions in more regions of the US, as well as through increased coverage of the treatment by government and other nonprivate insurance payers. Stereotactic body RT offers an efficacious treatment alternative with fewer fractions of RT, which may likely continue to drive its increased use for prostate cancer in the future. With the emergence of new technologies such as the now commercially available magnetic resonance–linear accelerators, treatment of localized prostate cancer with SBRT has never been more attractive for patients electing to pursue definitive RT. Given that a recent study demonstrated reduction in toxic effects of magnetic resonance imaging–guided SBRT compared with computed tomographic guidance, it will be interesting to see how or if new technology shifts patterns of practice moving forward.^20^

## Notes

Supplement 1. **eFigure.** Study Flow Diagram

Supplement 2.
Data Sharing Statement
