# Supplementary material for: Differential Use of Radiotherapy Fractionation Regimens in Prostate Cancer
Source: JAMA Netw Open. 2023 Oct 10;6(10):e2337165. doi: 10.1001/jamanetworkopen.2023.37165 (PMC10565603; doi:10.1001/jamanetworkopen.2023.37165)
Supplement: Supplement 1. — eFigure. Study Flow Diagram [file jamanetwopen-e2337165-s001.pdf]

Supplementary Online Content

Qureshy SA, Diven MA, Ma X, et al. Differential use of radiotherapy fractionation regimens in prostate cancer, 2004 to 2020. *JAMA Netw Open*. 2023;6(10):e2337165. doi:10.1001/jamanetworkopen.2023.37165

eFigure. Study Flow Diagram

This supplementary material has been provided by the authors to give readers additional information about their work.

© 2023 Qureshy SA et al. *JAMA Network Open*.

eFigure. Study Flow Diagram

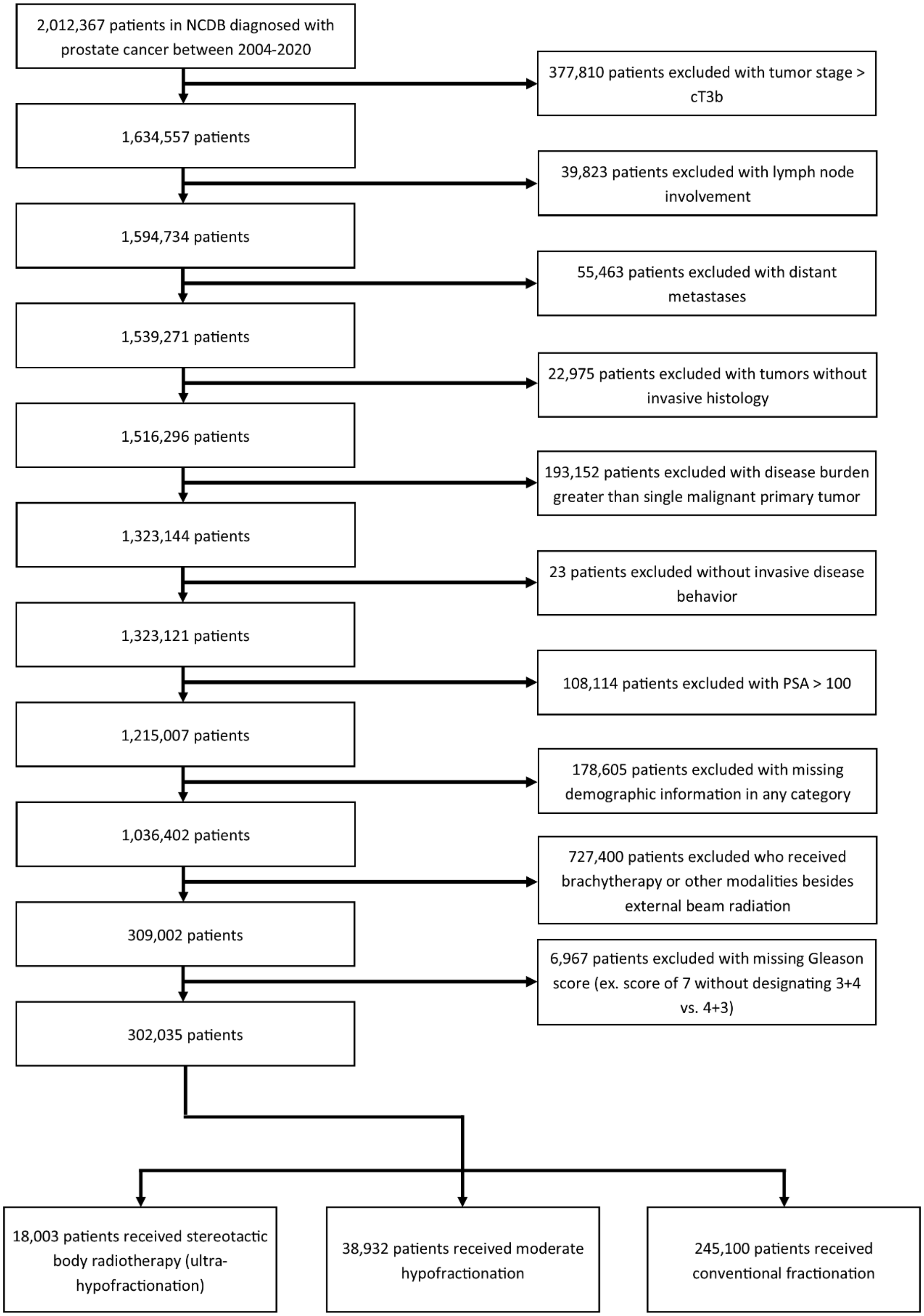

© 2023 Qureshy SA et al. *JAMA Network Open*.
