# Supplementary material for: Differential Use of Radiotherapy Fractionation Regimens in Prostate Cancer
Source: JAMA Netw Open. 2023 Oct 10;6(10):e2337165. doi: 10.1001/jamanetworkopen.2023.37165 (PMC10565603; doi:10.1001/jamanetworkopen.2023.37165)
Supplement: Supplement 2. — Data Sharing Statement [file jamanetwopen-e2337165-s002.pdf]

Data Sharing Statement

Qureshy. Differential Use of Radiotherapy Fractionation Regimens in Prostate Cancer. *JAMA Netw Open*. Published October 10, 2023. doi:10.1001/jamanetworkopen.2023.37165

Data
Data available: No

Additional Information
Explanation for why data not available: NCDB data base is controlled by ACS and is available upon request. Methods for our analysis would be available at request.
